# Molecular genetic studies and delineation of the oculocutaneous albinism phenotype in the Pakistani population

**DOI:** 10.1186/1750-1172-7-44

**Published:** 2012-06-26

**Authors:** Thomas J Jaworek, Tasleem Kausar, Shannon M Bell, Nabeela Tariq, Muhammad Imran Maqsood, Asma Sohail, Muhmmmad Ali, Furhan Iqbal, Shafqat Rasool, Saima Riazuddin, Rehan S Shaikh, Zubair M Ahmed

**Affiliations:** 1 Division of Pediatric Ophthalmology, Cincinnati Children’s Hospital Research Foundation, Cincinnati, OH, 45229, USA; 2 Institute of Molecular Biology & Biotechnology, Bahauddin Zakariya University, Multan, 60800, Pakistan; 3 Department of Ophthalmology, Nishter Hospital, Multan, Pakistan; 4 Department of Ophthalmology, College of Medicine, University of Cincinnati, Cincinnati, OH, 45229, USA; 5 Division of Pediatric Otolaryngology Head & Neck Surgery, Cincinnati Children’s Hospital Research Foundation, Cincinnati, OH, 45229, USA; 6 Department of Otolaryngology, College of Medicine, University of Cincinnati, Cincinnati, OH, 45229, USA

**Keywords:** TYR, OCA2, TYRP1, SLC45A2, SLC24A5, Pakistan, Exon-trapping, Oculocutaneous Albinism, Melanocytes, Hypopigmentation

## Abstract

**Background:**

Oculocutaneous albinism (OCA) is caused by a group of genetically heterogeneous inherited defects that result in the loss of pigmentation in the eyes, skin and hair. Mutations in the *TYR*, *OCA2*, *TYRP1* and *SLC45A2* genes have been shown to cause isolated OCA. No comprehensive analysis has been conducted to study the spectrum of *OCA* alleles prevailing in Pakistani albino populations.

**Methods:**

We enrolled 40 large Pakistani families and screened them for *OCA* genes and a candidate gene, *SLC24A5*. Protein function effects were evaluated using *in silico* prediction algorithms and *ex vivo* studies in human melanocytes. The effects of splice-site mutations were determined using an exon-trapping assay.

**Results:**

Screening of the *TYR* gene revealed four known (p.Arg299His, p.Pro406Leu, p.Gly419Arg, p.Arg278*) and three novel mutations (p.Pro21Leu, p.Cys35Arg, p.Tyr411His) in ten families. *Ex vivo* studies revealed the retention of an EGFP-tagged mutant (p.Pro21Leu, p.Cys35Arg or p.Tyr411His) tyrosinase in the endoplasmic reticulum (ER) at 37°C, but a significant fraction of p.Cys35Arg and p.Tyr411His left the ER in cells grown at a permissive temperature (31°C). Three novel (p.Asp486Tyr, p.Leu527Arg, c.1045-15 T > G) and two known mutations (p.Pro743Leu, p.Ala787Thr) of *OCA2* were found in fourteen families. Exon-trapping assays with a construct containing a novel c.1045-15 T > G mutation revealed an error in splicing. No mutation in *TYRP1*, *SLC45A2,* and *SLC24A5* was found in the remaining 16 families. Clinical evaluation of the families segregating either *TYR* or *OCA2* mutations showed nystagmus, photophobia, and loss of pigmentation in the skin or hair follicles. Most of the affected individuals had grayish-blue colored eyes.

**Conclusions:**

Our results show that ten and fourteen families harbored mutations in the *TYR* and *OCA2* genes, respectively. Our findings, along with the results of previous studies, indicate that the p.Cys35Arg, p.Arg278* and p.Gly419Arg alleles of *TYR* and the p.Asp486Tyr and c.1045-15 T > G alleles of *OCA2* are the most common causes of OCA in Pakistani families. To the best of our knowledge, this study represents the first documentation of *OCA2* alleles in the Pakistani population. A significant proportion of our cohort did not have mutations in known *OCA* genes. Overall, our study contributes to the development of genetic testing protocols and genetic counseling for OCA in Pakistani families.

## Introduction

Among the most visible phenotypic traits in humans is skin color. Loss of skin, hair and iris pigmentation, a condition known as oculocutaneous albinism (OCA), represents a significant load of human genetic diseases. OCA can manifest itself in syndromic and nonsyndromic forms under a variety of inheritance models [1]. At present, mutations in at least 16 loci have been causally linked with OCA [1,2]. More genes in humans are likely to be identified as implicated by new OCA syndromes [3,4]. Mutations at four loci, *OCA1* (*TYR*), *OCA2* (*OCA2*), *OCA3* (*TYRP1*) and *OCA4* (*SLC45A2*), have been shown to be necessary and sufficient to cause isolated OCA [1]. Approximately 450 different pathogenic alleles of these four genes have been documented (Human Gene Mutation Database (HGMD), http://www.hgmd.org/), and most of these sequence variations are located in the *TYR* gene.

Human chromosome 11q14.3 harbors the *TYR* gene (MIM# 606933), which encodes tyrosinase [5]. Tyrosinase is expressed in melanocytes and controls the biosynthesis of melanin from tyrosine at three levels [5]. To date, 291 pathogenic variants of the *TYR* gene have been identified in individuals with the OCA1 phenotype (HGMD). There is a presumptive genotype-phenotype correlation in which the severe pathogenic or null alleles of the *TYR* gene result in the OCA1A (MIM# 203100) phenotype, characterized by the loss of pigmentation in the skin, hair and eyes with translucent irises [6]. Hypomorphic alleles produce a spectrum of clinical phenotypes, known as OCA1B (MIM# 606952), which range from low to nearly normal levels of skin and hair pigmentation in adults.

*OCA2* (MIM# 203200) is located on human chromosome 15q11-q13 and has two non-coding and 23 coding exons. *OCA2* encodes a polypeptide of ~110 kDa with 12 putative transmembrane helices. As a member of the Na^+^/H^+^ antiporter family, the OCA2 protein is thought to play an essential role in maintaining the pH of the melanosomes, which regulates tyrosinase activity [7-10]. The OCA2 protein also participates in the sorting and transport of tyrosinase and tyrosinase-related protein 1 (TYRP1) to the plasma membrane [11-13]. *OCA2* mutations are the most common causes of OCA in Africa, with a prevalence rate as high as 1:3,900 being observed [14].

Human chromosome 9q23 harbors the *TYRP1* gene (MIM# 115501), which is known to cause OCA type 3 (MIM 203290; a.k.a Rufous OCA). The seven known coding exons of *TYRP1* (GenBank NM_000550) encode a tyrosinase-related protein of ~61 kDa with 41% sequence identity and 58% similarity to tyrosinase [15]. TYRP1 has partial tyrosinase hydroxylase activity and catalyzes the oxidation of 5,6-dihydroxyindole-2-carboxylic acid in the melanin biosynthesis pathway [16,17]. As of November 2012, only nine *TYRP1* mutations have been reported in the HGMD.

The OCA4 phenotype (MIM# 606574) is caused by mutations in the *SLC45A2* gene (MIM# 606202, a.k.a. *MATP*), which is located on human chromosome 5p13.3. In the HGMD, 76 pathogenic alleles of *SLC45A2* have been reported to date. In humans, seven known coding exons of *SLC45A2* transcribe four alternatively spliced variants. The longest spliced isoform (GenBank NM_016180) encodes a solute carrier family 45, member 2 (SLC45A2) protein composed of 530 amino acids and has a molecular weight of ~58 kDa. Although its precise function has not been elucidated, SLC45A2 probably acts as a melanosomal protein and substance transporter [18,19].

To the best of our knowledge, no comprehensive molecular analysis of these four known *OCA* genes has been conducted in Pakistani families segregating OCA. However, nine pathogenic variants of the *TYR* gene, including c.344delGA, p.Arg278*, p.Ser315_A316del, p.Gln328Glu, p.Glu376*, p.Gly419Arg, p.Pro431Thr, p.Pro431Leu and p.Glu453*, have been identified in mostly sporadic cases from Pakistan [20-25]. In addition to *TYR* alleles, only a single point mutation (c.1117 C > T, p.Arg373*) in *TYRP1* gene has been reported in a large consanguineous Pakistani family [25]. As a corollary of the inimitable socio-cultural customs in the population of Pakistan, approximately 60% of marriages are consanguineous, of which more than 80% are between first cousins [26]. These large consanguineous families are a powerful resource for genetic studies of recessively inherited disorders like OCA. In the present study, we analyzed the four *OCA* genes in 40 large Pakistani families to characterize the genetic lesion and to establish a mutational profile of the Pakistani albino population. In addition, we screened *SLC24A5* (MIM# 609802), which is responsible for ocular albinism and hypopigmentation in *Slc24a5* knockout mice and is known to regulate melanogenesis in humans [27,28]. The results of this study will be important for future diagnosis, genetic counseling, and molecular epidemiology of OCA.

## Materials and methods

### Family participation and clinical evaluation

This study was approved by the IRB Committees at the Children’s Hospital Research Foundation, USA (2010–0452) and the Institute of Molecular Biology & Biotechnology, Pakistan. Informed written consent was obtained from the adult subjects and the parents of minor subjects. Detailed clinical histories were obtained from participating family members and affected individuals were examined by an Ophthalmologist and a physician to rule out any obvious syndromic forms of OCA. Clinical features of OCA, such as hypopigmentation of the hair and skin and the presence of eye aberrations, including nystagmus, strabismus, photophobia and poor vision, were evaluated. Peripheral blood samples were collected from each participating individual for genomic DNA extraction.

### Mutational analysis

The primers used for PCR amplification and sequencing of the *TYR, OCA2, TYRP1, SLC45A2* and *SLC24A5* genes were designed using the Primer3 web site. The sequencing method for the PCR products has been described previously [29]. For specific amplification of exons 4 and 5 of *TYR*, we used the primers and PCR conditions described previously [30]. Briefly, the coding and noncoding exons of *TYROCA2TYRP1SLC45A2, SLC24A5* were PCR amplified from 50 ng genomic DNA, using ABI Veriti thermocyclers (Applied Biosystems, Austin, TX). PCR reactions (final volume 20 μl) were performed with genomic DNA in the presence of 5 pmol each of forward and reverse primers, 200 mM each dNTP, 1 x PCR buffer (GenSrcipt), 1.5–2.5 mM MgCl_2_ (GenScript), and 0.5 U of a thermostable DNA polymerase. For sequencing reaction, we added 3.2 pmol of primer, 0.2 μl of Big Dye Terminator Ready Reaction Mix (ABI Biosystems), and 2 μl of 5 x dilution buffer (400 mM Tris–HCl pH9 and 10 mM MgCl_2_). An ABI 3730xl DNA capillary sequencer was used to resolve the products, and Lasergene DNAstar software was used to analyze the results. Co-segregation of the mutations with OCA in each family was confirmed by sequencing. Control DNA samples from an ethnically matched Pakistani population were sequenced for mutant alleles of *TYR* and *OCA2*. Three prediction programs, Polyphen-2 [31], SNPs3D [32], and MutationTaster [33], were used to determine the effect of novel missense mutations. Effects of missense mutations on the structure of tyrosinase and OCA2 were also analyzed using the Project HOPE web server [34].

### Fluorescently tagged TYR expression constructs

The EGFP-tagged, full-length human *TYR* cDNA construct was generated using PCR primers located in exons 1 and 5. A retinal cDNA library (Clontech, Mountain View, CA) was used as the template source. The sequence-verified PCR product was inserted into the pEGFP-C2 vector (Clontech, Mountain View, CA). Constructs encoding the p.Pro21Leu, p.Cys35Arg and p.Tyr411His mutant forms of tyrosinase were prepared by site-directed mutagenesis (Agilent Technologies, Santa Clara, CA).

### Cell culture conditions, transfection and immunostaining

Human melanocyte cells were transiently transfected using Fugene-6 (Promega, Madison, WI) with 1.5 μg of the desired construct per well in a 6-well dish. After transfection, cells were incubated for 48 hours at either 37°C or 31°C followed by fixation with 4% paraformaldehyde. For visualization of the endoplasmic reticulum and early endosomes, anti-calregulin (Santa Cruz Biotechnology, Santa Cruz, CA) and anti-EEA1 (Abcam, Cambridge, MA) antibodies were used, respectively. A Zeiss LSM700 confocal microscope was used for imaging.

### Exon-trapping assay

To determine the effect of splice site mutation (c.1045-15 T > G) found in five OCA families, the wild-type and mutant exon 10 along with 200 bp flanking introns of *OCA*2 gene were PCR-amplified, cloned into the pSPL3 vector (Invitrogen, Carlsbad, CA), and sequence-verified. Purified, cloned DNA of the experimental, wild-type, and empty vector control constructs were separately transfected into COS-7 cells using the Fugene-6 reagent. Forty-eight hours after transfection of the pSPL3 constructs, RNA was extracted from the COS-7 cells using TRIzol reagent (Invitrogen, Carlsbad, CA), and single-stranded cDNA was synthesized (Clontech, Mountain View, CA). Primary PCR amplification of the cDNA was performed with vector primers. Ten microliters of each amplimer was analyzed on a 2% agarose gel. DNA bands were extracted and sequenced with the vector primers.

## Results

### Genetic and clinical analyses of *TYR* (*OCA1*)

**Figure 1:**
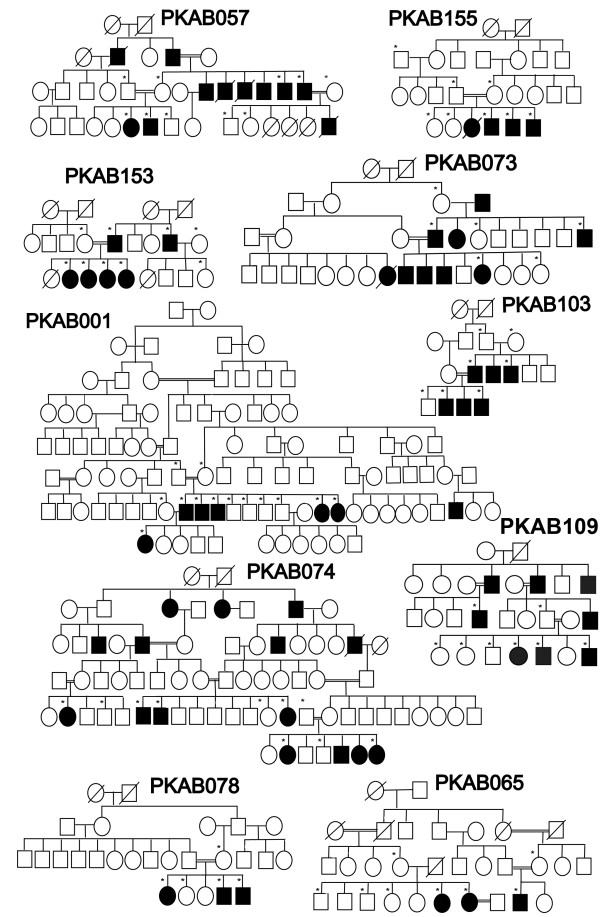
**Pedigrees of Pakistani families carrying* TYR *mutations.** Pedigrees of ten multi-generational families segregating recessive nonsyndromic OCA due to mutations in the *TYR* gene. Filled and empty symbols represent affected and unaffected individuals, respectively. Double lines indicate consanguineous marriages. Asterisks indicate subjects enrolled in the protocol that contributed DNA samples.

**Figure 2:**
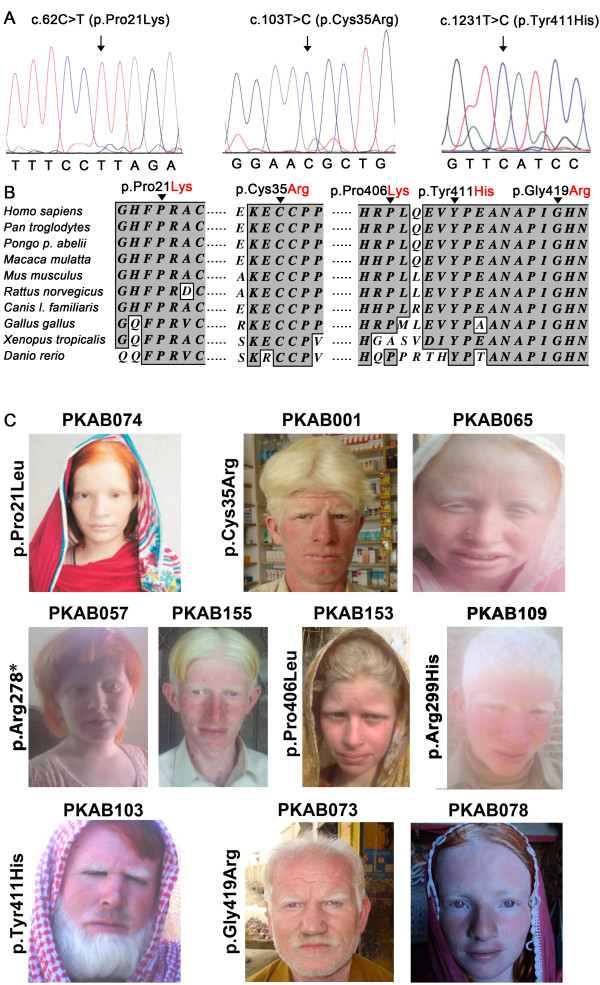
**Novel *TYR *mutations and resulting OCA1 phenotypes. A. **Electropherograms of amplimers from genomic DNA templates illustrating homozygosity for the substitution mutations found in the affected individuals of the families. Arrows indicate the site of the mutations. All of the mutations described here are numbered from the ATG start codon (GenBank NM_000372). **B**. Clustal W alignment of tyrosinase proteins from various species that shows the conservation of residues at positions 21, 35 and 411 among ten species. The conserved amino acids are shown with a dark gray background, and the nonconserved amino acids are shown with a white background. **C**. Photographs of ten OCA1 probands. The family number and the mutation identified in the *TYR* gene are given for each proband; some of the probands have used hair dyes.

Study subjects from forty families, segregating congenital onset, nonsyndromic, recessive OCA, were enrolled from different cities in the Punjab province of Pakistan. Sequence analysis of the *TYR* gene revealed seven probable pathogenic variants in ten of these families (Figure 1, Tables 1,2). Of these seven mutant alleles, four have been previously reported and include c.832 C > T (p.Arg278*), c.896A > G (p.Arg299His), c.1217 C > T (p.Pro406Leu) and c.1255 G > A (p.Gly419Arg) [5,20,24,35]. Three novel missense substitutions were identified including p.Pro21Leu (c.62 C > T), p.Cys35Arg (c.103 T > C) and p.Tyr411His (c.1231 T > C) (Figure 2A). All of these missense mutations affected amino-acid residues that are conserved among the tyrosinase orthologs (Figure 2B). We performed a haplotype analysis using eight closely linked single nucleotide polymorphisms (SNPs) in *TYR* to identify potential founder effects for the recurrent variants (p.Arg278*, p.Gly419Arg and p.Cys35Arg). Selection of these eight SNPs for haplotype analysis in Pakistani OCA families was based on the observance of high heterozygosity (> 0.3) in 32 control samples randomly collected from the Pakistani population. The results were consistent with common ancestors for each of the recurrent alleles in the Pakistani families studied here (data not shown). 

**Table 1:** **Mutant alleles of *TYR *found in ten Pakistani families**

**Nucleotide change**^**#**^	**Exon**	**Effect on protein**	Frequencies in control samples*	**Family**	**Ethnicity**	**Polyphen2**	**SNPs3D**	**Mutation Taster**	**Allele frequency in our OCA1 families**	**Known frequencies in other populations**	**References**
Missense											
**c.62 C > T**	1	p.Pro21Leu	0/380	PKAB074	Sayyed	Damaging	Damaging	Pathogenic	10%	N/A	This study
**c.103 T > C**	1	p.Cys35Arg	0/380	PKAB001 PKAB065	Malik Malik Jutt	Damaging	Damaging	Pathogenic	20%	N/A	This study
c.896A > G	2	p.Arg299His	0/372	PKAB109	Warraich	Damaging	Damaging	Pathogenic	10%	Caucasian 12.5%; Arab-Christian 1.6%, 2.6% and 3.3%; Chinese 18.75%; Indian 4.34%.	[10,36-40]
c.1217 C > T	4	p.Pro406Leu	0/372	PKAB153	Malik Jutt	Damaging	Damaging	Pathogenic	10%	Caucasian 2.94% and 25%; German 14.28%.	[37,41,42]
**c.1231 T > C**	4	p.Tyr411His	0/372	PKAB103	Arian	Damaging	Damaging	Pathogenic	10%	N/A	This study
c.1255 G > A	4	p.Gly419Arg	0/372	PKAB073 PKAB078	BhatiJutt	Damaging	Damaging	Pathogenic	20%	Caucasian 0.83%; Indo-Pakistan 25%; Pakistan 0.83%; Indian 4.34% and 20%; South-Indian 16.6%.	[20,36,37,43,44]
Nonsense											
c.832 C > T	2	p.Arg278*	0/372	PKAB057 PKAB155	Shaikh Rajpoot				20%	Guayanan 12.5%; Jewish 2.6%; Japanese 12.5%, 22.2% and 100%; European 2.5%; Mexican 0.83%; Indian 0.83% and 4.34%; Eastern Indian 8.3%, 25% and 100%; Syrian 0.83%; Chinese 18.75%.	[9,10,20,36,37,39,43-48]

^#^Novel mutations are in bold. *Frequencies were determined by sequencing at least 372 chromosomes from geographically and ethnically-matched subjects without any history of ocular disease. N/A: not applicable.

**Table 2:** **Clinical assessment of the affected individuals with mutations in *TYR *and *OCA2***

**Gene/Family**	**Mutation (protein)**	**Sex**	**Age (yrs)**	**Hair color**	**Skin color**	**Iris color**	**Visual Acuity Right Left**		**Type of refraction error**	**Fundus**	**Foveal hypoplasia**	**Photophobia**^**#**^	**Nystagmus**	**Con**
***TYR***														
PKAB074	p.Pro21Leu	M	19	Brown	Pinkish white	Grey/Brown	4/60	4/60	Myopic	Albinotic	Yes	Present	Yes	Yes
PKAB001	p.Cys35Arg	M	40	White	Pinkish white	Grey/Blue	6/38	6/38	Compound myopic hypermetropic astigmatism	Albinotic	Yes	Present	Yes	Yes
PKAB065	p.Cys35Arg	F	28	White	White^a^	Grey/Blue	N/A	N/A	N/A	N/A	N/A	Present	Yes	No
PKAB057	p.Arg278*	F	7	White	White^a^	Light Brown	N/A	N/A	N/A	N/A	N/A	Present	Yes	Yes
PKAB155	p.Arg278*	M	16	White	White^a,b^	Grey/Blue	N/A	N/A	N/A	N/A	N/A	Present	Yes	Yes
PKAB109	p.Arg278*	M	24	N/A	N/A	N/A	N/A	N/A	N/A	N/A	N/A	Present	Yes	Yes
PKAB153	p.Pro406Leu	F	18	Yellow	White^a^	Light Grey	N/A	N/A	N/A	N/A	N/A	Present	Yes	Yes
PKAB103	p.Tyr411His	M	30	White	Pinkish White	Grey/Blue	6/60	6/60	Compound hypermetropic	Albinotic	Yes	Present	Yes	No
PKAB073	p.Gly419Arg	M	45	White	White^a^	Grey/Blue	N/A	N/A	N/A	N/A	N/A	Present	Yes	No
PKAB078	p.Gly419Arg	F	12	White	White^a,b^	Grey/Blue	N/A	N/A	N/A	N/A	N/A	Present	Yes	Yes
***OCA2***														
PKAB052	p.Asp486Tyr	F	1.5	White	White	Grey/Blue	N/A	N/A	N/A	N/A	N/A	Present	Present	Yes
PKAB054	p.Asp486Tyr	F	13	Yellowish- White	White^a,b^	Blue	6/60	6/60	Compound Myopic	Albinotic	Hypoplasia	Present	Present	Yes
PKAB055	p.Asp486Tyr	F	1.5	White	White	Light Grey	N/A	N/A	N/A	N/A	N/A	Present	Present	Yes
PKAB067	p.Asp486Tyr	M	35	White	White^a^	Grey/Blue	N/A	N/A	N/A	N/A	N/A	Present	Present	No
PKAB101	p.Asp486Tyr	M	25	White	White^a^	Grey/Blue	6/60	6/60	Mixed Astigmatism	Albinotic	Hyoplasia	Present	Present	Yes
PKAB063	p.Met318Ile p.Leu527Arg	F	5	White	White^a^	Grey/Blue	N/A	N/A	N/A	N/A	N/A	Present	Present	Yes
PKAB058	p.Pro743Leu	M	30	White	White^a^	Grey/Blue	1/60	4/60	Hypomyopic	Albinotic	N/A	Present	Present	No
PKAB072	p.Pro743Leu	M	6	White	White^a^	Grey/Blue	N/A	N/A	N/A	N/A	N/A	Present	Present	Yes
PKAB071	p.Ala787Thr	M	10	Yellowish-White	White^a,b^	Blue	Fc3	Fc3	Compound Hypermetropic	Albinotic	Hypoplasia	Present	Present	Yes
PKAB60	c.1045-15 T > G	F	6	White	White^a,b^	Grey/Brown	6/36	6/36	Hypermetropic Astigmatism	Albinotic	Hypoplasia	Present	Present	Yes
PKAB068	c.1045-15 T > G	F	7	Yellowish-White	White^a^	Blue/Brown	N/A	N/A	N/A	N/A	N/A	Present	Present	Yes
PKAB079	c.1045-15 T > G	M	19	Yellowish-White	Reddish	Grey/Brown	N/A	N/A	N/A	N/A	N/A	Present	Present	Yes
PKAB151	c.1045-15 T > G	F	6	White	White^a^	Grey/Blue	N/A	N/A	N/A	N/A	N/A	Present	Present	Yes
PKAB152	c.1045-15 T > G	F	12	N/A	N/A	N/A	N/A	N/A	N/A	N/A	N/A	Present	Present	Yes

^#^All individuals show squinting in normal sunlight. ^a^Reddish spots throughout the skin and lips appeared sun damaged. ^b^Show blistering on exposed skin and generalized sunburn redness. *N/A* not available, *Cons* consanguineous union.

All of the affected individuals from the ten families had nystagmus and photophobia, regardless of their sex and age (Figure 2C, Table 2 and Additional file 1: Table S1). Inter-familial variation of hair color was noted among individuals, ranging from white to honey blonde or brown (Figure 2C, Table 2 and Additional file 1: Table S1). Notably, reddish spots and marked sun-damage on the skin and grossly enlarged veins in the cheeks and lips were observed (Figure 2C).

### Functional analysis of novel missense alleles of tyrosinase

Three prediction programs, specifically, Polyphen-2 [31], SNPs3D [32], and MutationTaster [33], suggested that each of the three new missense mutations were deleterious (Table 1). We also used the HOPE prediction program [34] to assess further the effect of the missense mutations on the secondary structure of the encoded protein. Both the p.Pro21 and p.Cys35 residues are located in the amino terminal region. Due to their charge, size, and hydrophilic properties, the amino acids lysine and arginine at positions 21 and 35, respectively, were predicted to disrupt protein topology, which could result in protein misfolding. Also, p.Tyr411 is located close to a defined copper-binding site within the luminal domain; inserting histidine at this position was predicted to cause an empty space in the core of the protein and the loss of hydrophobic interactions, because of the smaller size and hydrophilicity of histidine.

**Figure 3:**
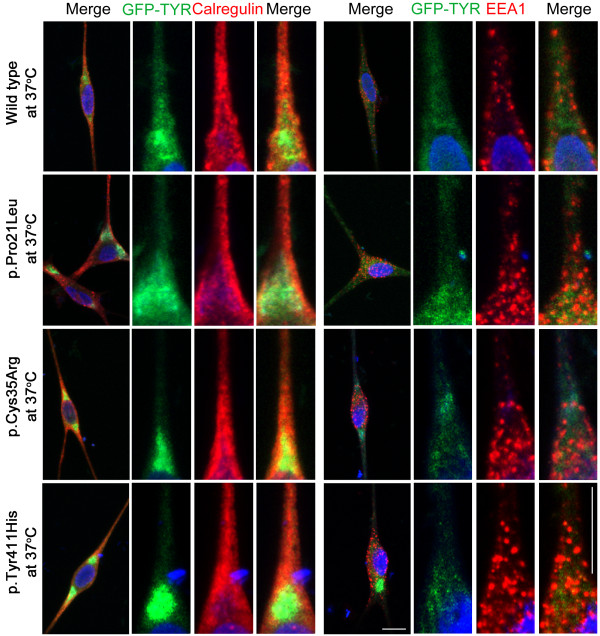
**Subcellular distribution of wild-type and mutant tyrosinase in human melanocytes grown at 37°C.** Subcellular distribution of wild-type and mutant (p.Pro21Leu, p.Cys35Arg and p.Tyr411His) tyrosinase proteins (green) in transiently transfected human melanocytes grown at 37°C. Calregulin and EEA1 were used as markers for the endoplasmic reticulum (red) and the early endosome (red), respectively. For each construct, boxed regions were magnified in the adjacent panels. Merged images show the co-localization of the p.Pro21Leu, p.Cys35Arg and p.Tyr411His tyrosinase mutations with calregulin, which indicates ER retention. The scale bar represents 10 μm for all panels.

**Figure 4:**
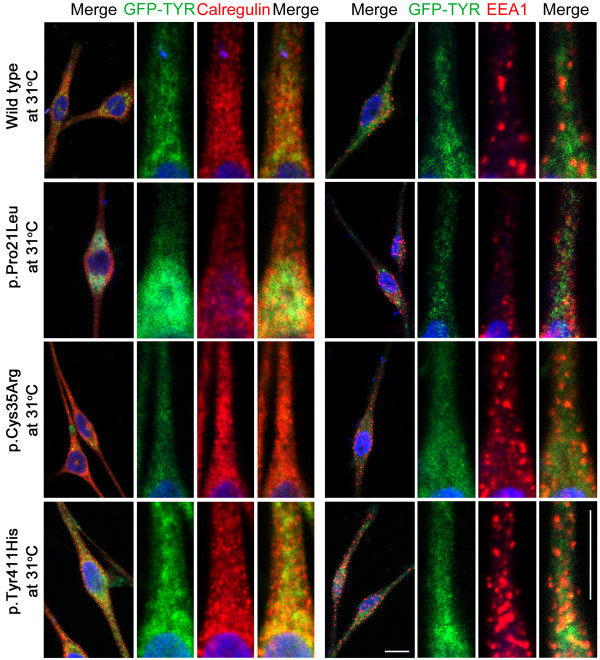
**Subcellular distribution of wild-type and mutant tyrosinase in human melanocytes grown at 31°C.** The subcellular localization of GFP-tyrosinase in wild-type and p.Pro21Leu-transfected cells was not significantly different when cells were incubated at 31°C or at 37°C. After transfection with either p.Cys35Arg or p.Tyr411His mutant constructs, the melanocytes grown at 31°C showed an increase in the cytoplasmic vesicular co-localization of the mutant protein with EEA1. For each construct, boxed regions were magnified in the adjacent panels. The scale bar represents 10 um for all panels.

Generally, missense alleles of the *TYR* gene result in the retention of the encoded mutant protein in the endoplasmic reticulum (ER) [49]. To determine the effect of the three novel missense mutations (p.Pro21Leu, p.Cys35Arg and p.Tyr411His) on the localization of tyrosinase, we transiently transfected human melanocytes with GFP-tagged, full-length wild-type and mutant *TYR* cDNA constructs (Figure 3). Wild-type tyrosinase was localized predominantly throughout the cytoplasm of melanocytes with some expression in the ER (Figure 3). The low expression of wild-type tyrosinase in the ER might be due to newly synthesized polypeptides that are retained in the ER by chaperones until they are properly folded and assembled [50]. Immunofluorescence studies with calregulin (an ER marker) and EEA1 (an early endosome marker) demonstrated that the mutant proteins predominantly co-localized with calregulin, indicating retention in the ER (Figure 3). A portion of the known human and mouse *TYR* mutations, especially those present in the copper-binding region, have shown temperature-sensitive behavior [5,35,51-54]. Therefore, we also tested the effect of temperature on the localization of wild-type and mutant tyrosinase proteins by growing transfected melanocytes at 37°C and 31°C (Figures 3 and 4). Interestingly, a decrease in temperature resulted in an increase in the cytoplasmic vesicular co-localization of the p.Cys35Arg and p.Tyr411His mutant protein with EEA1 (Figure 4). Melanocytes that were transfected with wild-type and p.Pro21Leu constructs and grown at 31°C, did not show any significant change in the localization pattern relative to cells grown at 37°C (Figure 4). 

### Frequency of the *rs1042602* cSNP in the Pakistani population

**Figure 5:**
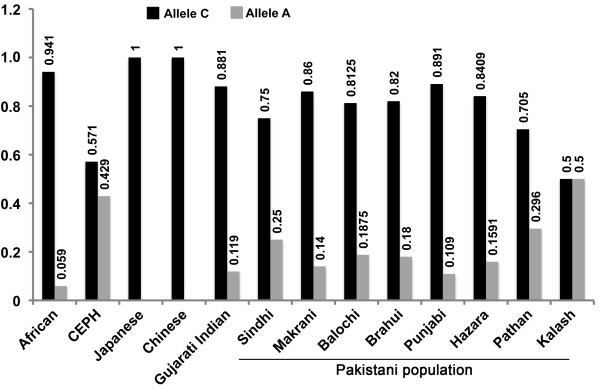
**Allele frequency of the *rs1042602 *cSNP in the Pakistani population.** The distribution of an ancestral C and derived A allele of *TYR* among Pakistani population. DNA samples from 200 individuals belonging to various ethnic groups within province of Punjab, Pakistan were genotyped for *rs1042602*. Also shown is the Human Genome Diversity Project data for comparison [55]. Details are available at the HGDP website (http://hgdp.uchicago.edu/).

Previous studies have shown a biased distribution of the *TYR* cSNP, p.Ser192Tyr (*rs1042602*) among the various populations studied in the International HapMap Project (http://hapmap.ncbi.nlm.nih.gov/). To determine the minor allele frequency (MAF) of the *rs1042602* cSNP in the Punjab province of Pakistan, we genotyped 200 unrelated, normal individuals from different ethnic groups (Figure 5). Significant variation in the frequency of c.575 C > A in different regions of Pakistan was observed (Figure 5 and Additional File 2: Figure S1).

All of the families in our study were enrolled from the Punjab province of Pakistan, and five of the forty families that were screened for *TYR* had a c.575 C > A (p.Ser192Tyr) polymorphism. In two of these five families, we did not find any other mutation in *TYR*, except for the heterozygous c.575 C > A. We found that c.575 C > A in these two families did not co-segregate with the OCA phenotype. Thus, we did not consider p.Ser192Tyr to be a pathogenic variant.

### Genetic and clinical analyses of *OCA2*

**Figure 6:**
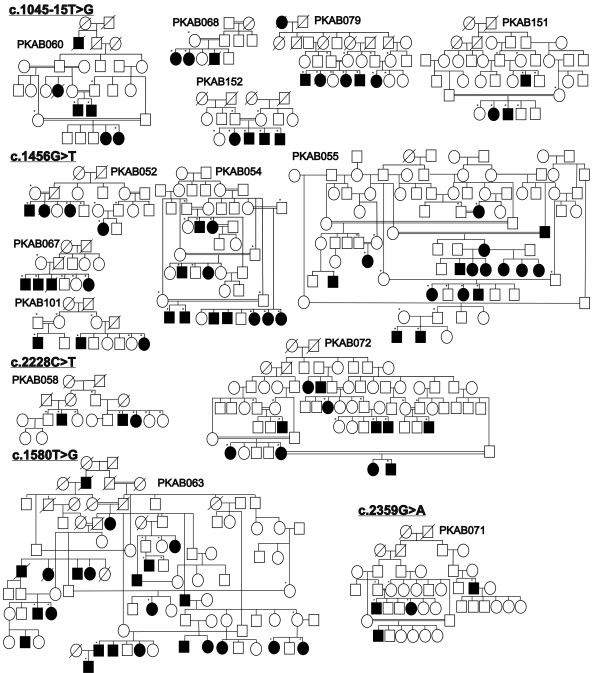
**Pedigrees of Pakistani families segregating *OCA2 *mutations.** Pedigrees of fourteen multi-generational families with mutations identified in the *OCA2* gene. Filled and empty symbols represent affected and unaffected individuals, respectively. Double lines indicate consanguineous marriages. Asterisks indicate subjects enrolled in the protocol that contributed DNA samples.

**Figure 7:**
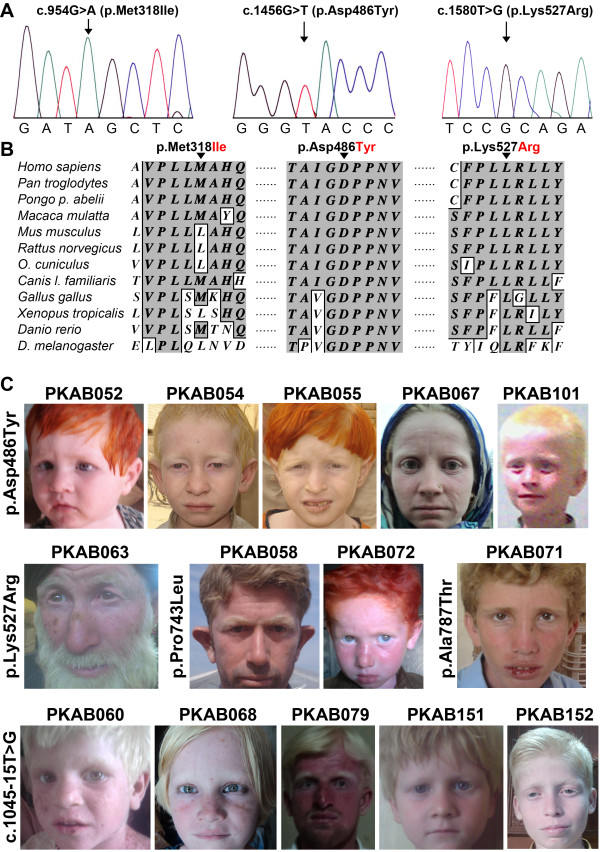
**Novel *OCA2 *mutations and resulting OCA2 phenotypes. A.**Electropherograms of amplimers from genomic DNA templates illustrating homozygosity for the substitution mutations found in the affected individuals of the families. Arrows indicate the site of the mutations. All mutations described here are numbered from the ATG start codon (GenBank NM_000275). **B**. Clustal W alignment of OCA2 proteins from various species shows conservation of the residues at positions 318, 486 and 527 among twelve species. The conserved amino acids are shown with a dark gray background, and the nonconserved amino acids are shown with a white background. **C**. Photographs of fourteen OCA2 probands. The family number and the mutation identified in the *OCA2* gene are given for each proband; a number of the probands shown have used hair dyes.

Sequence analysis of the *OCA2* gene revealed six variants in fourteen families (Figures 6 and 7, Table 3), including c.954 G > A, p.Met318Ile; c.1045-15 T > G; c.1456 G > T, p.Asp486Tyr; c.1580 T > G, p.Lys527Arg; c.2228 C > T, p.Pro743Lys; and c.2359 G > A, p.Ala787Thr. Three different prediction algorithms were used to determine the effects of the missense mutations identified in our cohort. Three of the four missense alleles were predicted to be damaging by all three programs (Table 3). The fourth change, c.954 G > A (p.Met318Ile), was predicted to be a benign polymorphism (Table 3). Furthermore, ClustalW alignment of the OCA2 proteins from eleven different species showed that the methionine residue at position 318 is not conserved and in fact, mouse, rat, rabbit, frog, and Drosophila have a leucine at this position, which is an amino acid closely related to isoleucine (Figure 7). In contrast, the remaining two residues (p.Asp486 and p.Lys527) mutated in other families are highly conserved in the eleven species analyzed here (Figure 7). Although not found in the DNA samples from 200 ethnically matched control individuals, p.Met318Ile segregated in cis with another missense mutation (p.Lys527Arg) of OCA2 in the PKAB063 family, thereby preventing us from exploring its functional role in OCA2 pathogenesis. 

**Table 3:** **Mutations of *OCA2 *segregating in Pakistani families**

**Nucleotide change**^#^	**Frequency in control samples**	**Effect on protein**	**Location**	**Family**	**Ethinicity**	Haplotype* S1-S2-S3-S4-S5-S6-S7-S8	**Polyphen 2**	**SNPs3D**	**Mutation Taster**	**Allele frequency this study**	**Regulatory region sequence**^**¥**^
Missense											
**c.954 G > A**	0/200	p.Met318Ile	loop TM2-3	PKAB063	Warraich	C-C-C-G-A-T-G-A	Benign	Benign	Polymorphism	7.14%	TAA**A**TG
**c.1456 G > T**	0/344	p.Asp486Tyr	within TM7	PKAB052	Lanjay	T-C-T-A-A-C-A-G	Damaging	Damaging	Pathogenic	35.71%	TAA**G**TG
				PKAB054	Mehay	T-C-T-A-A-C-A-G					TAA**G**TG
				PKAB055	Mehay	T-C-T-A-A-C-A-G					TAA**G**TG
				PKAB067	Ghallu	T-C-T-A-A-C-A-G					TAA**G**TG
				PKAB101	Chaaki	T-C-T-A-A-C-A-G					TAA**A**TG
**c.1580 T > G**	0/298	p.Leu527Arg	within TM8	PKAB063	Warraich	C-C-C-G-A-T-G-A				7.14%	TAA**A**TG
c.2228 C > T		p.Pro743Leu	loop TM12-13	PKAB058	Arain	C-C-T-G-A-C-A-A	Damaging	Damaging	Pathogenic	14.30%	TAA**A**TG
				PKAB072	Joyia	C-C-T-G-A-C-A-A					TAA**A**TG
c.2359 G > A		p.Ala787Thr	within TM13	PKAB071	Chohan	C-C-C-G-A-C-G-A	Damaging	Damaging	Pathogenic	7.14%	TAA**A**TG
											
Splice site											
**c.1045-15 T > G**	0/364	splicing	within TM3	PKAB060	Bubar	C-C-T-G-A-T-G-A	Damaging	Damaging	Pathogenic	35.71%	TAA**A**TG
		error^§^		PKAB068	Sindhu Jutt	C-C-T-G-A-T-G-A					TAA**A**TG
				PKAB079	Abbasi	C-C-T-G-A-T-G-A					TAA**A**TG
				PKAB151	Ansari	T-C-T-G-A-T-G-A					TAA**A**TG
				PKAB152	Ansari	C-C-T-G-A-T-G-A					TAA**A**TG

^#^Given in bold are the novel variants found in this study. *SNPs used for haplotyping: S1, rs17565841; S2, rs12592307 (p.S788S); S3, rs1800411 (p.C517C); S4, rs1900758; S5, rs1800410; S6, rs10852218; S7, rs1800404; S8, rs12913832. According to Feb. 2009 UCSC Human Genome browser assembly (GRCh37/hg19), the S2 to S6 are present within the coding (S2 and S3) or intronic regions of *OCA2*. ^§^Human Splicing Finder program (http://www.umd.be/HSF/) predicted a cryptic splice donor site 12 nucleotide upstream of known splice acceptor site for exon 9 due to the mutation. ^¥^Conserved regulatory element sequence present within intron 86 of the *HERC2* gene. *N/A* not applicable. *TM* transmembrane domain. TMpred-Prediction of transmembrane regions and orientation (http://www.ch.embnet.org/software/TMPRED_form.html) was used for OCA2 domain prediction.

Five of the OCA2 families segregated a c.1045-15 T > G change, and five other families had a c.1456 G > T (p.Asp486Tyr) mutation (Figure 6 and Table 3). We performed haplotype analyses of eight closely linked short tandem repeats and single nucleotide polymorphisms to identify potential founder effects for recurrent variants of the *OCA2* gene. The results are consistent with common ancestors for each of the two alleles (Table 3).

There was no obvious genotype-phenotype correlation observed in the affected individuals harboring different *OCA2* gene alleles (Table 2 and Additional file 1: Table S1). The affected individuals from the fourteen families had nystagmus and photophobia, regardless of their sex and age (Figure 7C, Table 2 and Additional file 1: Table S1). Most of the affected individuals had white or yellowish white hair color (Figure 7C, Table 2 and Additional file 1: Table S1). Inter-familial variation in iris color was noted, with tones ranging from light grey to blue-brown (Table 2). Similar to OCA1 families, the presence of reddish spots and marked sun-damage on the skin with grossly enlarged veins in the cheeks and lips was observed. Some of the affected individuals were observed to use hair dyes to color their hair (Figure 7C, Table 2 and Additional file 1: Table S1).

Although there were no pathogenic mutations in the *OCA2* promoter or in the cis-regulating element present in intron 86 of the *HERC* gene regulatory region [56], we found the blue eye color-associated allele (TAA**G**TG) of SNP *rs12913832* in five of the OCA2 families (Table 3). In these five families, the *rs12913832* “G” allele was found to be in linkage disequilibrium with the c.1456 G > T (p.Asp486Tyr) mutation in the *OCA2* gene (Table 3). Affected individuals of these five families had grayish-blue or blue eye color (Table 2 and Additional file 1: Table S1). The presence of the blue eye color allele of SNP *rs12913832* might contribute to the OCA phenotype by reducing the expression of the mutated OCA2 protein in these affected individuals [56].

### Effect of the c.1045-15 T > G mutation on splicing

**Figure 8:**
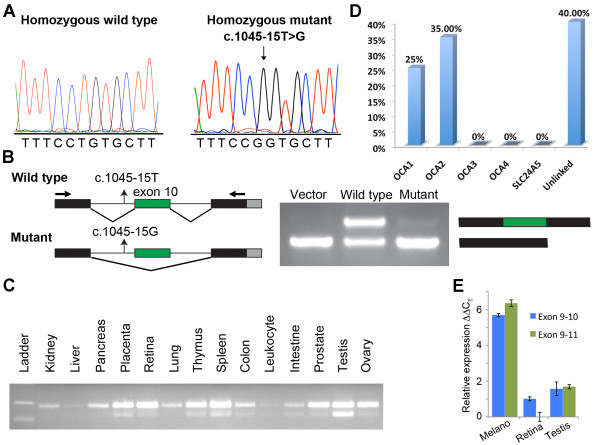
**Functional analysis of c.1045-15 T > G mutation. A**. Electropherograms of amplimers from genomic DNA templates illustrating homozygosity for the wild-type and c.1045-15 T > G substitution mutation found in the affected individuals of the five OCA2 families. The arrow indicates the site of the mutation. **B**. To determine the effect of the c.1045-15 T > G mutation on splicing, exon 10 with 200 bp of the flanking intron of *OCA2* was introduced into the pSPL3 vector and analyzed through an *in vitro* splicing assay. The transfected empty pSPL3 vector produced the expected product of 177 bp, whereas the wild-type exon 10 splice site produced two bands with (249 bp) and without (177 bp) exon-10 splicing when amplified with vector primers, which might indicate the presence of weak splice junctions around exon 10. The construct with the c.1045-15 T > G mutation produced a band of 177 bp, which upon sequencing, revealed the skipping of exon 10. With the mutant construct, a weak band of ~249 bp was occasionally observed, but sequencing revealed an aberrant splice product. **C**. Human *OCA2* isoforms with and without exon 10 are expressed in many tissues. **D**. Molecular genetic analysis of known OCA genes in a cohort of forty Pakistani families indicates that (a) OCA2 mutations are the most common cause of OCA, and (b) a significant number of families do not have mutations in the known OCA genes. **E**. Real-time quantitative RT–PCR analysis of *TYR* mRNAs level in human melanocytes, retina and testis cDNA libraries. C_T_ is the observed threshold number of PCR cycles required for detection of the amplification product; ΔC_T_ is the calculated difference in C_T_ between the *TYR* gene and an internal control standard (*GAPDH*) measured in the same sample. ΔΔC_T_ is the calculated difference in ΔC_T_ between the experimental and exon 9–11 isoform in retina. Compared to the retina and testis, melanocytes have a relatively high expression of both exon 9–10 and exon 9–11 isoforms of *TYR*.

To determine if c.1045-15 T > G (Figure 8A) alters the normal splicing of *OCA2* mRNA, we made two constructs of genomic DNA for exon trapping. One construct was for the wild-type genomic sequence spanning intron 10 through intron 11, and the other had the splice site mutation c.1045-15 T > G (Figure 8B). The transfected empty pSPL3 vector produced the expected product of 177 bp, whereas the wild-type exon 10 splice site produced two bands, one with (249 bp) and one without (177 bp) exon 10 splicing, when amplified with vector primers (Figure 8B), which might indicate the presence of weak splice junctions around exon 10. When transfected in the exon trapping system, the construct with the mutated 5’ splice site (c.1045-15 T > G) produced a band of 177 bp that had exon 10 spliced out (Figure 8B). Although a weak band of ~249 bp was occasionally observed, sequencing revealed an aberrant spliced product. The results from the exon-trapping assay demonstrate that the c.1045-15 T > G mutation results in an mRNA that skips exon 10. If only exon 10 is skipped, then there is a deletion of twenty-four amino acids, thereby resulting in the loss of the third transmembrane domain of the full-length protein. However, analysis of known *OCA2* transcripts indicated that exon 10 is alternatively spliced in most human tissues (Figure 8C). Quantitative real-time PCR analysis indicated that the *OCA2* transcript including exon 10 is more abundant in human retina compared to transcripts without exon 10 (Figure 8E).

### Genetic analysis of *TYRP1*, *SLC45A2* and *SLC24A5* genes

Although we did not screen the unknown regulatory regions, sequencing of all the non-coding and coding exons of the *TYRP1*, *SLC45A2* and *SLC24A5* genes in the 40 families segregating nonsyndromic OCA did not reveal any obvious pathogenic mutations (Figure 8D).

## Discussion

In this study, we identified seven different pathogenic variants, three of which are novel, as the cause of OCA1 in ten Pakistani families. Two of the novel missense mutations, p.Pro21Leu and p.Cys35Arg, replaced highly evolutionarily conserved amino acid residues. The p.Cys35Arg mutation was found in two families, and SNP analysis revealed a common haplotype harboring this allele, which indicates a founder effect. The other common alleles found in the OCA1 families were p.Gly419Arg and p.Arg278* (Table 2). Both of these variants have been previously found in other Pakistani families [23,25,35]. Although no genotype data are available from the previous studies, our SNP analysis revealed common haplotypes in families sharing the same mutations. Our findings, along with the results of previous studies, indicate that p.Cys35Arg, p.Arg278* and p.Gly419Arg are the three most common mutations causing OCA1 in Pakistani families [23,25,35].

Interestingly, two of the novel variants, p.Cys35Arg and p.Tyr411His, might have temperature-sensitive behavior that could be due to a subtle conformational defect or gross protein misfolding [57]. A phenotypic evaluation of individuals homozygous for this allele did not reveal a temperature-sensitive phenotype. The loss of pigmentation in exposed skin areas was not strikingly different than that in less-exposed skin areas (e.g., legs, chest or abdomen); this finding could be due to the effect of the hot local climate (37°C - 52°C) on melanocyte growth and melanogenesis in the skin [58].

The association of the *rs1042602* cSNP of *TYR* with squamous cell carcinoma of the skin in Caucasians and with pigmentation variation in the south Asian population has been documented [59,60]. This cSNP results in the substitution of serine with tyrosine at position 192 (p.Ser192Tyr) within the first copper-binding site (CuA) of tyrosinase [61]. Enzymatic analyses have revealed an approximately 40% reduction in the catalytic activity of tyrosinase, due to the p.Ser192Tyr mutation [61]. Interestingly, the distribution of the *rs1042602* cSNP alleles varies significantly among different individuals from different geographical origins within Pakistan and thus provides a useful marker for epidemiological studies (Additional file 2: Figure S1).

In the cohort of Pakistani families segregating OCA studied here, fourteen out of forty families have mutations in *OCA2*. These results indicate that OCA2 is more prevalent than OCA1 in Pakistan in contrast to the Indian population [36,62]. Of the six distinct mutations in the *OCA2* gene, three (p.Asp486Tyr, p.Leu527Arg, c.1045-15 T > G) have not been found in any of the various ethnic populations analyzed to date; therefore, they may be specific to Pakistani albino individuals. Of the two known mutations, p.Pro743Leu was previously identified in individuals of Caucasian, African-American and European ancestry [37,63-65], whereas the second mutation, p.Ala787Thr, was initially identified in the Chinese population [66]. Another missense mutation, p.Ala787Val, affecting the same codon of the *OCA2* gene as p.Ala787Thr, was also reported to cause oculocutaneous albinism [67], further confirming the necessity of the alanine residue at this position for proper OCA2 protein function.

Affected individuals of five OCA2 families were homozygous for the c.1045-15 T > G mutation. Although the *in vivo* effects of c.1045-15 T > G are not known, this mutation is expected to produce only the *OCA2* isoform without exon 10, which is predicted to encode a protein with no third transmembrane domain. Therefore, the level of normal OCA2 full-length protein required for the transport of tyrosinase to the plasma membrane might be affected by c.1045-15 T > G and thus cause OCA.

We found inter-familial variation in the clinical phenotype among the families segregating the same alleles of *TYR* or *OCA2* (Table 2 and Additional file 1: Table S1). However, no obvious genotype-phenotype correlation was observed. A significant overlap in the range of phenotypes in individuals with *TYR* (OCA1) and *OCA2* mutations was found, which makes genetic screening obligatory for the diagnosis of the type of albinism of the affected individuals. We compared the frequencies of all mutant *TYR* and *OCA2* alleles among our forty OCA families. Mutations in exons 1, 2 and 4 combined accounted for all of the mutant alleles of the *TYR* gene in our cohort (Table 1). For the *OCA2* gene, mutations in exons 10 and 14 collectively accounted for ~67% (10/15) of mutant alleles. Taken together, hierarchical mutation screening of these five exons of *OCA* genes in the nonsyndromic albino Pakistani population might reveal pathogenic alleles in approximately 43% (95% confidence interval: 28.5 - 57.9%) of cases and would be a cost-effective approach for molecular diagnosis.

Mutations in the protein coding exons or in the splice junctions of *TYROCA2TYRP1* and *SLC45A2* and *SLC24A5* were not found in 16 families. There are at least three possible reasons to explain these findings. First, cryptic mutations might be present in the regulatory or splicing elements of these genes. Presently, we do not know the locations of the regulatory elements of these genes. Secondly, although family clinical histories and evaluation suggested no other clinical phenotype besides OCA, some of these families might have syndromic OCA. Currently, mutations in at least 12 loci have been causally linked with syndromic OCA [1,2]. There may also be an additional gene in which mutant alleles cause nonsyndromic OCA. Several previous studies of the four known *OCA* genes revealed no mutations in some of the affected individuals screened [36,68,69]. A need clearly exists for further genetic examination of this disease, as does the opportunity to understand this complex disorder more clearly.

## Conclusions

Our results show that twenty-four families harbor twelve mutations (six previously reported and six novel mutations), but 40% of the ascertained OCA families had no apparent pathological mutations in the known *OCA* genes. Although our sample size was not large enough, based on our results, it is tempting to speculate that *OCA2* mutations are more prevalent than *OCA1* mutations in the Pakistani population. Nevertheless, this information would be useful for future diagnosis, genetic counseling and molecular epidemiology of OCA in the Pakistani population.

## Competing interests

The authors declare that they have no competing interests.

## Authors’ contributions

Z.M.A. and R.S.S conceived and designed the study; T.J.J. performed RT-PCR, mutational analyses, cloned isoforms, performed transfection studies and provided bioinformatic evaluations; T.K., N.T., M.I.M., A.S., F.I. and Sh.R. enrolled families and obtained clinical data; S.M.B. performed mutational screening; R.S.S and M.A. supervised the work at the Institute of Molecular Biology and Biotechnology, Multan; Z.M.A. and S.R. supervised the work at CCHMC. T.J.J. and Z.M.A. wrote the manuscript; All authors read and approved the final manuscript.

## Supplementary Material

Additional file 1 **Table S1. **Clinical assessment of the affected individuals with mutations in *TYR* and *OCA2.* Click here for file

Additional file 2 **Figure S1.** Schematic and geographical representation of allele frequency of the *rs1042602* cSNP in the Pakistan. The distribution of an ancestral C (black) and derived A allele (gray) of *TYR* among Pakistani population.^#^All individuals show squinting in normal sunlight. ^a^Reddish spots throughout the skin and lips appeared sun damaged. ^b^Show blistering on exposed skin and generalized sunburn redness. Cons: consanguineous union. Click here for file
