# Supplementary material for: Molecular genetic studies and delineation of the oculocutaneous albinism phenotype in the Pakistani population
Source: Orphanet J Rare Dis. 2012 Jun 26;7:44. doi: 10.1186/1750-1172-7-44 (PMC3537634; doi:10.1186/1750-1172-7-44)
Supplement: Additional file 1 — Table S1. Clinical assessment of the affected individuals with mutations in TYR and OCA2. [file 1750-1172-7-44-S1.doc]

***Supplementary Table 1: Clinical assessment of the affected individuals with mutations in TYR and*** OCA2

| **Gene / Family** | **Mutation**  **(protein)** | **Sex** | **Age**  **(yrs)** | **Hair color** | **Skin color** | **Iris color** | **Photophobia#** | **Nystagmus** | **Con** |
| --- | --- | --- | --- | --- | --- | --- | --- | --- | --- |
| ***TYR*** |  |  |  |  |  |  |  |  |  |
| PKAB074 | p.Pro21Leu | M  F  M | 13  14  19 | White  White  Yellow | Pinkish White  Pinkish White  Pinkish White | Grey/Brown  Brown  Grey/Brown | Present  Present  Present | Yes  Yes  Yes | Yes  Yes  Yes |
| PKAB001 | p.Cys35Arg | F  M  M  M | 11  25  37  41 | White  White  White  White | Pinkish White  Pinkish White  Pinkish White  Pinkish White | Grey/Blue  Grey/Blue  Grey/Blue  Grey/Blue | Present  Present  Present  Present | Yes  Yes  Yes  Yes | Yes  Yes  Yes  Yes |
| PKAB065 | p.Cys35Arg | M  F | 6  28 | White  White | Whitea­  Whitea | Grey/Blue  Grey/Blue | Present  Present | Yes  Yes | Yes  Yes |
| PKAB057 | p.Arg278* | F | 7 | White | Whitea | Light Brown | Present | Yes | Yes |
| PKAB155 | p.Arg278* | M | 16 | White | Whitea,b | Grey/Blue | Present | Yes | Yes |
| PKAB109 | p.Arg278* | M  M  M | 22  24  45 | White  White  White | Whitea,b  Whitea,b  Pinkish White | Grey/Blue Grey/Blue  Grey/Blue | Present  Present  Present | Yes  Yes  Yes | Yes  Yes  Yes |
| PKAB153 | p.Pro406Leu | F  F  F  M  M | 2.5  7  14  45  55 | Yellow  Yellow  Yellow/Silver  Yellow  White | Pinkish Whitea  Pinkish Whitea  Pinkish Whitea  Pinkish Whitea  Pinkish Whitea | Grey/Blue  Grey/Blue  Grey/Blue  Light Grey  Grey/Blue | Present  Present  Present  Present  Present | Yes  Yes  Yes  Yes  Yes | Yes  Yes  Yes  No  No |
| PKAB103 | p.Tyr411His | M  M | 30  38 | White  White | Pinkish White  Pinkish White | Grey/Blue  Grey/Blue | Present  Present | Yes  Yes | No  No |
| PKAB073 | p.Gly419Arg | M | 40 | White | Whitea | Grey/Blue | Present | Yes | No |
| PKAB078 | p.Gly419Arg | M  F  M | 6  10  12 | White  White  White | Whitea,b  Whitea,  White | Grey/Blue  Grey/Blue  Grey/Blue | Present  Present  Present | Yes  Yes  Yes | Yes  Yes  Yes |
| ***OCA2*** |  |  |  |  |  |  |  |  |  |
| PKAB052 | p.Asp486Tyr | F  M | 1.5  22 | White  White | White  Pinkish White | Grey/Blue  Grey/Blue | Present  Present | Yes  Yes | Yes  Yes |
| PKAB054 | p.Asp486Tyr | M  M  F | 6  12  13 | Yellow-White  Yellow-White  Yellow-White | Whitea,b  Whitea,b  Whitea,b | Blue  Grey/Blue  Blue | Present  Present  Present | Yes  No  Yes | Yes  Yes  Yes |
| PKAB055 | p.Asp486Tyr | F  F  F  M | 1.5  5  8  25 | White  White  White  White | White  White  White  White | Light Grey  Grey/Blue  Grey/Blue  Grey/Blue | Present  Present  Present  Present | Yes  No  Yes  No | Yes  Yes  Yes  Yes |
| PKAB067 | p.Asp486Tyr | F  M  M | 20  32  35 | Yellow-White  Yellow-White  White | Whitea  Whitea  White | Grey/Blue  Grey/Blue  Grey/Blue | Present  Present  Present | Yes  Yes  Yes | Yes  Yes  Yes |
| PKAB101 | p.Asp486Tyr | M  M | 6  25 | White  White | Whitea  White | Grey/Blue  Grey/Blue | Present  present | Yes  Yes | Yes  Yes |
| PKAB063 | p.Met318Ile  p.Leu527Arg | F  F  F  M | 5  7  12  55 | White  White  White  White | Whitea  Whitea  Whitea  Whitea | Grey/Blue  Grey/Blue  Grey/Brown  Grey/Brown | Present  Present  Present  Present | Yes  Yes  Yes  Yes | Yes  Yes  Yes  Yes |
| PKAB058 | p.Pro743Leu | F  M  M | 24  30  50 | Yellow-White  White | Whitea  White  White | Grey/Blue  Grey/Blue  Grey/Blue | Present  Present  Present | Yes  Yes  Yes | Yes  Yes  Yes |
| PKAB072 | p.Pro743Leu | M  M  M  M | 4  6  7  12 | Yellow-White  White  Yellow-White  White | Pinkish.Whitea  White  Pinkish White  Pinkish White | Grey/Blue  Grey/Blue  Grey/Blue  Grey/Blue | Present  Present  Present  Present | Yes  Yes  Yes  Yes | Yes  Yes  Yes  Yes |
| PKAB071 | p.Ala787Thr | F  M | 10  14 | Yellow  Yellow-White | Pinkish Whitea,b  White | Grey/Blue  Grey/Blue | Present  Present | Yes  Yes | Yes  Yes |
| PKAB060 | c.1045-15T>G | M  F  F | 3  4  6 | Yellow  White  White | Whitea,b  Whitea,b  Whitea,b | Grey/Brown  Grey/Brown  Grey/Brown | Present  Present  Present | Yes  Yes  Yes | Yes  Yes  Yes |
| PKAB068 | c.1045-15T>G | F  F | 7  7 | Yellow-White  Yellow-White | Whitea  Whitea | Blue/Brown  Blue/Brown | Present  Present | Yes  Yes | Yes  Yes |
| PKAB079 | c.1045-15T>G | F  F  M  M | 5  15  17  19 | Yellow-White  Yellow  Yellow  Yellow | Pinkish White  Pinkish White  Pinkish White  Reddish | Grey  Grey  Grey  Grey/Brown | Present  Present  Present | Yes  Yes  Yes  Yes | Yes  Yes  Yes  Yes |
| PKAB151 | c.1045-15T>G | M  F  M | 1  6  19 | White  White  Brown | Whitea  Pinkish White  Pinkish White | Grey/Blue  Grey/Blue  Grey/Blue | Present  Present  Present | Yes  Yes  No | Yes  Yes  No |
| PKAB152 | c.1045-15T>G | M  M  M | 1.5  6  12 | Yellow-White  Yellow-White  Yellow-White | Whitea  White  White | Grey/Blue  Grey/Blue  Grey/Brown | Present  Present Present | Yes  Yes  Yes | Yes  Yes Yes |

#All individuals show squinting in normal sunlight. aReddish spots throughout the skin and lips appeared sun damaged. bShow blistering on exposed skin and generalized sunburn redness. Cons: consanguineous union
