# Supplementary figures and images for: Molecular genetic studies and delineation of the oculocutaneous albinism phenotype in the Pakistani population
Source: Orphanet J Rare Dis. 2012 Jun 26;7:44. doi: 10.1186/1750-1172-7-44 (PMC3537634; doi:10.1186/1750-1172-7-44)

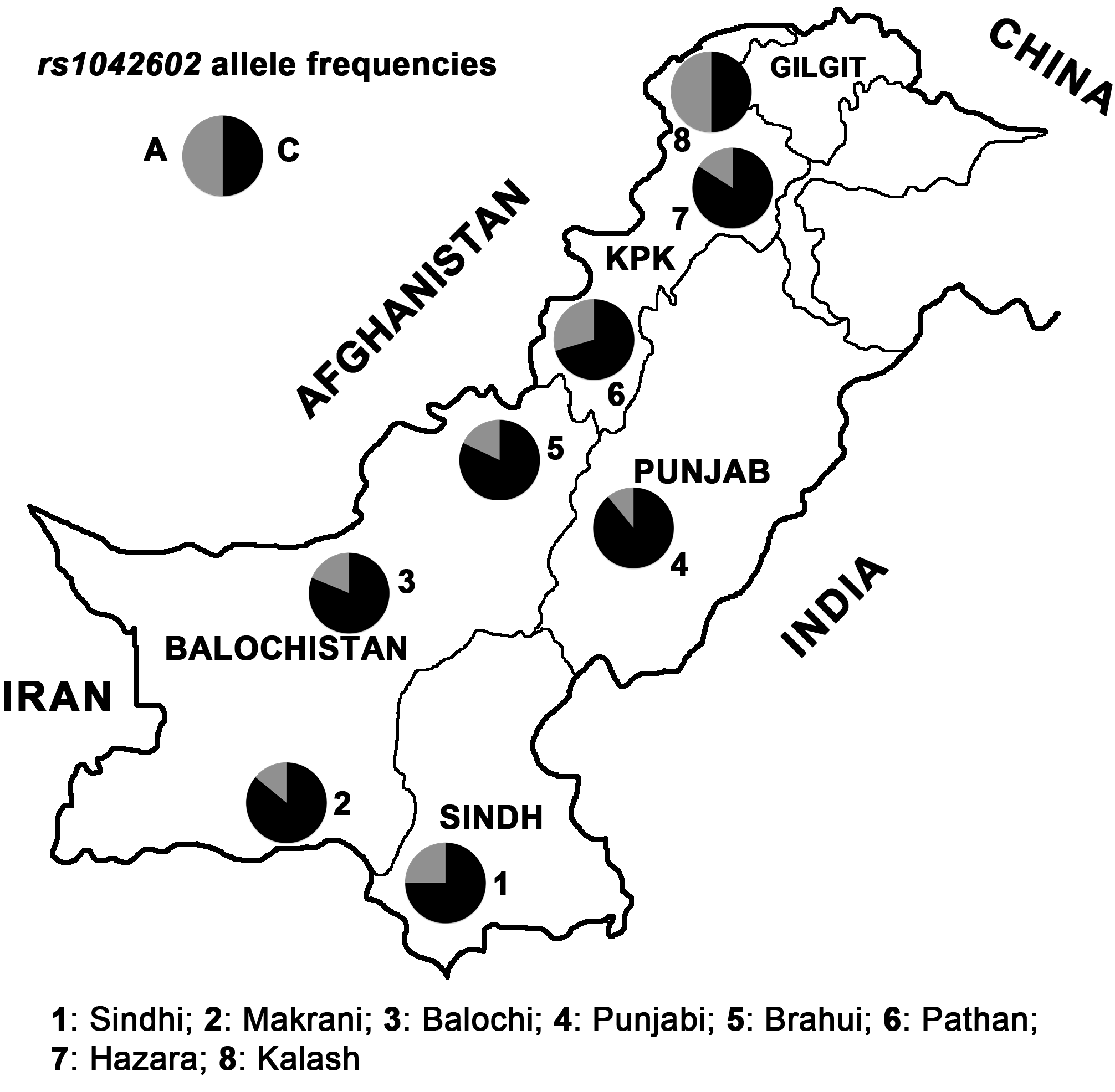

Supplement: Additional file 2 — Figure S1. Schematic and geographical representation of allele frequency of the rs1042602 cSNP in the Pakistan. The distribution of an ancestral C (black) and derived A allele (gray) of TYR among Pakistani population.#All individuals show squinting in normal sunlight. aReddish spots throughout the skin and lips appeared sun damaged. bShow blistering on exposed skin and generalized sunburn redness. Cons: consanguineous union. [file 1750-1172-7-44-S2.tiff]
